# Supplementary material for: Causal interplay between lactose intolerance and gut microbiota: a combined bidirectional Mendelian randomization and in vivo validation study
Source: Front Nutr. 2026 Jun 1;13:1803337. doi: 10.3389/fnut.2026.1803337 (PMC13265576; doi:10.3389/fnut.2026.1803337)
Supplement: Supplementary file 1 [file Data_Sheet_1.zip › supplementary materials/Forward/forest plot/ebi-a-GCST90027737.finngen_R12_E4_LACTONAS.pdf]

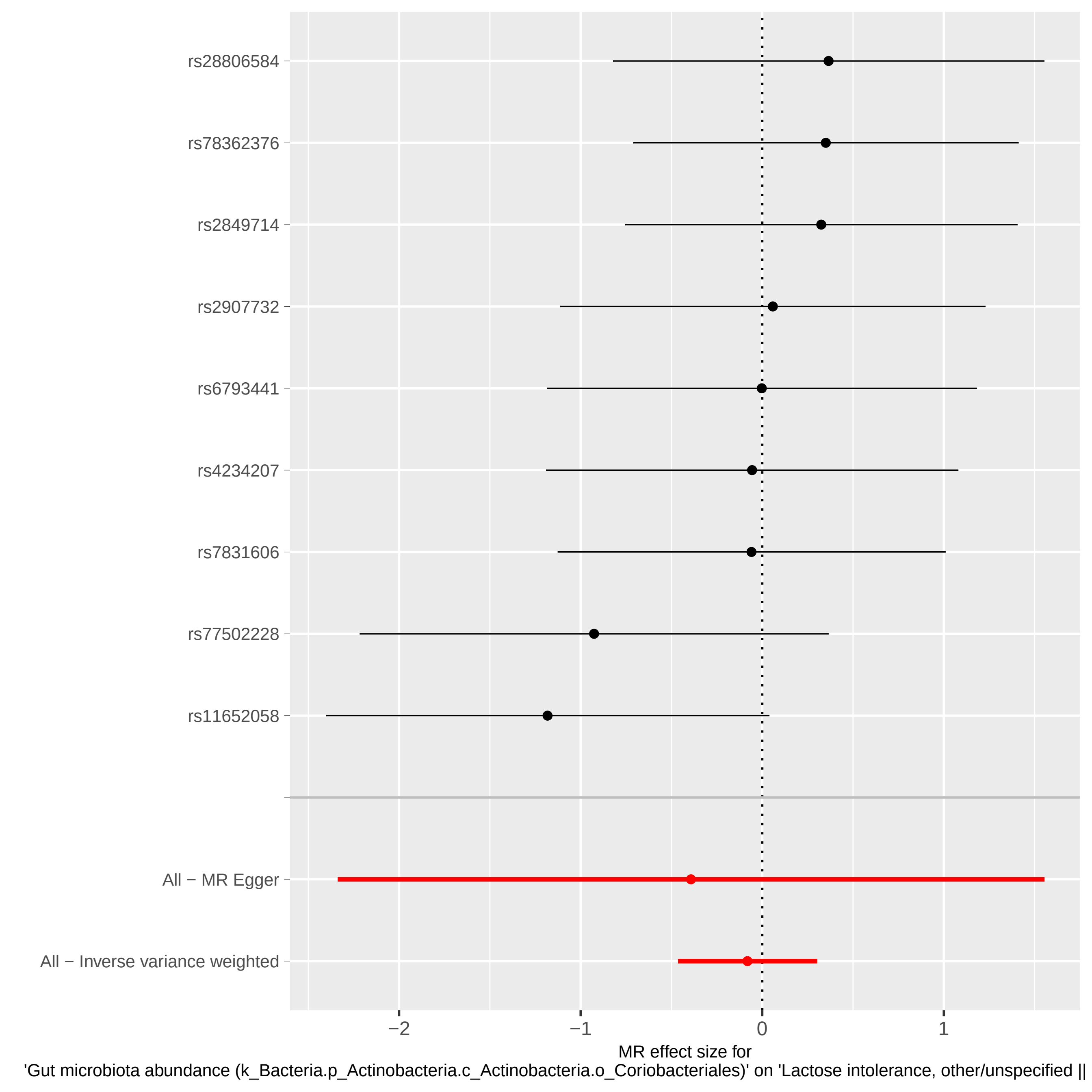

rs28806584
rs78362376
rs2849714
rs2907732
rs6793441
rs4234207
rs7831606
rs77502228
rs11652058
All − MR Egger
All − Inverse variance weighted
−2
−1
0
1
MR effect size for
'Gut microbiota abundance (k_Bacteria.p_Actinobacteria.c_Actinobacteria.o_Coriobacteriales)' on 'Lactose intolerance, other/unspecified ||
